# Implementation of psycho-existential symptom distress screening among Italian healthcare providers

**DOI:** 10.1017/S1478951525101302

**Published:** 2025-12-29

**Authors:** Andrea Bovero, Giorgia Feni, Laura Valenti, Alessandro Valle, Massimo Di Maio, Ernesta Audisio, Silvia Varani, Irene Di Girolamo, David Kissane, Luca Ostacoli, Francesca Cotardo

**Affiliations:** 1 Clinical Psychology Unit, A.O.U. Città della Salute e della Scienza, Turin, Italy; 2 Fondazione FARO Onlus, Turin, Italy; 3 Department of Oncology, University of Turin, A.O.U. Città della Salute e della Scienza di Torino, Turin, Italy; 4 Complex Structure of Hematology, A.O.U. Città della Salute e della Scienza, Turin, Italy; 5 Fondazione ANT, National Tumor Assistance (ANT), Bologna, Italy; 6 Department of Clinical and Biological Sciences, University of Turin, Orbassano, Turin, Italy; 7 University of Notre Dame, Sydney, NSW, Australia; 8 Faculty of Medicine, Nursing and Health Sciences, Monash University, Melbourne, VIC, Australia; 9 Department of Psychology, University of Turin, Turin, Italy; 10 Psychology Unit, A.S.L. TO5, Turin, Italy

**Keywords:** Existential distress, symptom assessment, palliative care, hopelessness, demoralization

## Abstract

**Objectives:**

Existential distress is a debilitating condition in end-of-life cancer patients. The Psycho-existential Symptom Assessment Scale (PeSAS) was developed to screen psycho-existential symptoms in palliative care, but limited research has examined its use. This study aimed to implement the Italian version of the PeSAS in palliative care services and to evaluate changes in healthcare providers’ (HCPs) competence after experiential training. It also aimed to estimate the frequency of psycho-existential symptoms and explore the scale structure using network analysis.

**Methods:**

Two-hour experiential workshops were conducted in 5 Italian palliative care services by a clinical psychologist specialized in psycho-oncology and palliative care. Training covered psycho-existential distress, role-play, and feedback. Pre- and post-workshop questionnaires assessed clinicians’ self-efficacy in evaluating physical, psycho-existential, and suicidal symptoms, managing distress, and providing psychosocial support. Patient cross-sectional data were analyzed with descriptive statistics, *t*-tests, chi-square tests, and exploratory graph analysis.

**Results:**

One hundred one clinicians from 3 services participated. Significant results were found in HCPs’ self-efficacy, with the largest effect in assessing suicidal symptoms (Cohen’s *d* = 0.54), followed by managing distress (*d* = 0.47) and evaluating psycho-existential symptoms (*d* = 0.40). Of 210 patients screened, 194 were included. PeSAS scores were strongly associated with Hopelessness (strength = 1.30) and depression (1.18), while being trapped by illness (−1.64) and wishing to die (−1.12) had weaker associations.

**Significance of results:**

The Italian PeSAS is feasible for integration into palliative care. Strong associations highlight targets for interventions, while weaker associations suggest the need for additional approaches. PeSAS enhances HCPs’ ability to address the psycho-existential needs in end-of-life care.

## Introduction

Existential distress is a complex, debilitating condition experienced by patients facing severe life-threatening illness, such as cancer (Vehling and Kissane 2018). It represents a central multidimensional phenomena, including physical, psychological, and spiritual aspects, that can compromise quality of life (Vehling and Kissane 2018; Fay and OBoyle 2019; Kissane et al. 2022; Bovero et al. 2023b). According to the palliative care approach, which aims to improve the quality of life, systematic assessment of psycho-existential needs is a crucial component of comprehensive care (Sawatzky et al. 2016; Wachholtz et al. 2016; Bovero et al. 2018; Fay and OBoyle 2019; Kissane et al. 2022; WHO 2023). Existential distress reflects the emotional impact of losses and life changes caused by cancer, as well as the struggle to maintain autonomy during progressive loss of control (Kissane et al. 2022; Bovero et al. 2024).

The existential component of psychological distress is highly prevalent, especially among end-of-life cancer patients, compared to cancer outpatients and the general population (Bovero et al. 2019; Ann-Yi and Bruera 2022). Frequency rates vary depending on the assessment tools. In a large cohort of advanced cancer patients, Philipp et al. (2025) found clinically significant existential distress in 46.4% of patients, including dignity-related distress (38.7%), death anxiety (27.3%), and demoralization (12.5%). Mental disorders were present in 26.2% of patients, with 20% showing co-occurrence of existential distress and psychiatric conditions. Bovero et al. (2018) identified existential distress as a clinically significant problem for 18.8% of the patients, while Rattner (2022) found that over 60% experienced moderate to severe distress related to loss of meaning and dignity. These findings underscore the need for screening tools to identify and address psycho-existential distress in palliative care.

The literature suggests that early and systematic screening of psychological symptoms and existential difficulties supports HCPs to identify patients at higher risk, affecting outcomes for patients and caregivers (Philipp et al. 2021; Bovero et al. 2023a). A multidimensional approach is needed to better understand suffering, monitor symptom progression and treatment effectiveness, and guide clinical strategies (Kissane et al. 2022; Kissane 2024).

Several validated psychotherapeutic approaches have been developed to support end-of-life cancer patients in managing existential distress, including Meaning-Centered Group Psychotherapy, Individual Meaning-Centered Psychotherapy, dignity therapy (DT), and managing cancer and living meaningfully (Chochinov et al. 2005; Breitbart et al. 2015; Rodin et al. 2018; Terao and Satoh 2022). Despite their efficacy, addressing existential distress remains challenging for HCPs. Insufficient training and education programs are key barriers that may affect HCPs’ ability to implement comprehensive screening and management of psycho-existential distress in their routine clinical practice (Knies et al. 2019).

The Psycho-Existential Symptom Assessment Scale (PeSAS), developed by Kissane et al. (2022), is a validated and effective screening tool for psycho-existential distress in palliative care. Based on the Edmonton Symptom Assessment Scale by Bruera and colleagues (Bruera et al. 1991), the PeSAS scale evaluates the presence of 10 symptoms related to psycho-existential well-being. Patients rate each symptom on a numerical scale from zero (absent) to 10 (severe).

Following Kissane et al. (2023), symptom intensity was obtained by summing the scores of each item, with higher scores (greater than or equal to 8) related to clinically significant distress. This standardized approach allows clinicians to assess the level of distress feasibly in routine care.

Given the limited research on psycho-existential distress screening tools, the primary aim of this study was to assess the implementation of the PeSAS in the Italian palliative care context. We introduced the tool through experiential training workshops designed to enhance HCPs’ ability to assess psycho-existential distress in end-of-life cancer patients. Secondary aims were to estimate the frequency of symptoms with PeSAS and to explore its structure using network analysis.

## Methods

The research protocol was designed to ensure a rigorous implementation of the PeSAS in Italian palliative care. It comprised a 4-stage framework: 1. translation and back-translation following international guidelines for cultural adaptation of patient-reported outcome measures (Beaton et al. 2000). This process involved a multidisciplinary panel of experts who reviewed all versions to ensure semantic, conceptual, and cultural equivalence; 2. development of a structured workshop to introduce HCPs to the PeSAS and its clinical application. The design of this workshop followed the model proposed by Kissane et al. (2022). Pre- and post-workshop assessments evaluated changes in participants’ knowledge, confidence, and clinical approaches to assessing existential distress in end-of-life cancer patients; 3. interactive role-playing sessions with participants alternating roles between physician and patient to enhance practical skills. These sessions used predefined clinical scenarios focusing on different patient characteristics; 4. trained participants applied PeSAS in real-world palliative care clinical settings.

### Ethical approval

Ethical approval of the study was obtained from the Hospital’s and Hospice’s Ethics Committee (#0034403). All participants included in the study were informed about the aims of the research and provided informed written consent.

### Sample and setting

This study reports the introduction of PeSAS screening through 5 experiential workshops in 5 palliative care services in Turin.

Inclusion criteria for HCPs were active employees in palliative care settings (hospitals, hospices, or home services) during data collection; participation in a 2-hour training workshop about the use of the PeSAS tool; and completion of both pre- and post-workshop questionnaires.

Detailed information on the clinicians trained, classified by professional and sociodemographic characteristics, is provided in Table 1; services are deidentified.

**Table 1.** Clinicians’ sociodemographic characteristics by discipline and across contributory services, showing distribution of disciplines

Characteristics	Consultants physicians	Trainee physicians	Nurses	Psychologists	Healthcare assistants	Physiotherapists
Age ranges						
% 20–29 years	0.0	71.4	23.5	5.56	12.5	0.0
% 30–49 years	45.5	14.3	35.3	55.6	37.5	100.0
–% >50 years	54.5	14.3	41.2	38.9	50.0	0.0
Clinical experience						
Mean in years (range)	17.1 (1–34)	1.86 (1–3)	16.5 (1–42)	12 (1–25)	7.9 (2–20)	15.0 (1–15)
Religion %						
% Christian	60.6	14.3	44.1	61.1	62.5	0.0
% Atheist	27.3	71.4	35.3	22.2	25.0	100.0
% Other	12.1	14.3	20.6	16.7	12.5	0.0
Country of birth						
% Italian	90.9	85.7	88.2	100.0	50.0	100.0
% Other	9.1	14.3	11.3	0.0	50.0	0.0
Clinician profile for de-identified services						
Service A	3	4	0	0	0	0
Service B	3	0	6	1	3	1
Service C	6	1	6	0	0	0
Service D	9	2	12	13	5	0
Service E	12	0	10	4	0	0
Total	33	7	34	18	8	1

Inclusion criteria for the patients were: age ≥18; cancer diagnosis; ability to provide informed consent; and eligibility for palliative care (National Law on Palliative Care and Pain Treatment, No. 38/2010). This required a terminal illness with no available or appropriate curative treatment, an unfavorable prognosis with life expectancy ≤4 months, and a Karnofsky Performance Status ≤50. Consistent with Kissane et al. (2022), an Australian Modified Karnofsky Performance Status (AKPS) score ≤50 was also required.

## Procedures

### Training

Two of our training workshops were conducted by a clinical psychologist specialized in psycho-oncology and palliative care. Each included an introduction to psycho-existential distress and the PeSAS tool, role-play, and feedback.

HCPs were trained to obtain symptom scores from their patients in 2–3 minutes, identify and summarize the 2 or 3 highest scores, and respond empathetically to reported distress. Participants were advised to seek permission to discuss scores with the multidisciplinary team and refer for additional psychosocial support.

### Introduction of screening

Screening was implemented across service components as patients were admitted and integrated as part of routine clinical assessment. After patients were informed about the study and provided consent, an HCP administered the Italian version of PeSAS (see Supplementary material).

### Data collection

Data were collected from HCPs’ pre- and post-workshop questionnaires. Pre-workshop data included sociodemographic and professional characteristics of the sample. The pre-workshop questionnaire also included the evaluation of HCPs’ baseline knowledge of psycho-existential topics and their assessment in palliative care (on a 10-point Likert scale ranging from zero “not confident” to 10 “highly confident”). The post-workshop questionnaire included the same 6 questions rating perceived self-efficacy in assessing physical, psycho-existential, and suicidal symptoms, empathic responsiveness, managing distress, and facilitating psychosocial support (on a 10-point Likert scale ranging from 0 “not effective” to 10 “highly effective”). It also evaluated participants’ perception of the program’s usefulness through 3 specific questions on acquired skills (from 0 “no improvement” to 10 “highest improvement”), addressing an increase in knowledge about existential suffering, the ability to recognize it, and perceived improvement in their ability to provide high-quality care. Services were deidentified as A–E.

### Data analysis

We analyzed de-identified and aggregate data to assess changes in efficacy before and after training workshops. The frequency of symptoms was calculated using aggregate patient data, with measurement precision evaluated by comparing 95% confidence intervals. Mean scores differences before and after the workshops were tested using *t*-tests, while frequency distributions were tested using chi-square tests. Statistical significance was set at *p* < 0.05 (2-tailed).

Exploratory graph analysis (EGA) using the EGAnet package in R 4.0.0 (R Core Team 2020) was conducted to explore the internal structure of PeSAS items. This method employed a Gaussian Graphical Model, estimating partial correlations between items while controlling for all others. The resulting network was visualized as a graph, with nodes representing symptoms and edges (green lines, intensity, and thickness) representing partial correlations. Centrality indices of strength, closeness, and betweenness were calculated to assess their importance. Visualization was performed using the Fruchterman–Reingold algorithm (Epskamp et al. 2018).

## Results

### Clinicians

Between 2023 and 2024, 101 clinicians participated in training sessions across 3 healthcare services and 2 workshops. Professional backgrounds and sociodemographic characteristics are presented in Table 1.

Data presented in Table 2 showed significant improvements in self-perceived efficacy in the administration of PeSAS scale among HCPs. Confidence in assessing suicidal symptoms was the largest improvement (*d* = 0.54), followed by managing overall distress (*d* = 0.47) and evaluating psycho-existential symptoms (*d* = 0.40).

**Table 2.** Clinicians’ confidence scores out of 10 pre- and post-training in administering the Psycho-Existential Symptom Assessment Scale (PeSAS) for 101 clinicians who attended the 2-hour workshop

Skills confidence	Pre-training mean (SD)	Post-training mean (SD)	Change in means	Cohen’s *d*^a^
Assess physical symptoms	7.5 (1.49)	7.79 (1.51)	0.29	0.19
Assess psycho-existential symptoms	6.94 (1.40)	7.52 (1.54)	0.58	0.40
Assess suicidal symptoms	5.30 (2.28)	6.53 (2.27)	1.23	0.54
Empathic responsiveness	7.69 (1.64)	7.86 (1.60)	0.17	0.10
Manage overall distress	6.63 (1.64)	7.39 (1.59)	0.76	0.47
Effect referrals when needed	7.68 (1.43)	7.9 (1.51)	0.22	0.20

SD = standard deviation.

a Cohen’s *d*: 0.2 = small effect size, 0.5 = medium effect size, 0.8 = large effect size.

By analyzing the 3 specific questions included in the final section of the post-workshop questionnaire, improvements were observed in multiple domains following the training. The mean (SD) values were 7.09 (2.05) for knowledge of existential suffering, 7.02 (2.02) for ability to recognize it, and 6.89 (2.19) for perceived improvement in patient care.

### Services

Services A and C operated within a predominantly medical–nursing model, whereas Service F had a broader interdisciplinary composition. Services B and E employed a mixed-clinician model, while Service D followed a psychosocial approach. The implementation of the PeSAS was flexible and gradual, guided by clinicians’ judgment and each service’s organization. As clinicians’ confidence increased, its integration into routine clinical practice became more frequent and seamless.

### Patients

A total of 210 end-of-life cancer patients were eligible to participate in the study.

Six patients declined after reading the PeSAS items, 8 died before the start of the study, and 2 lacked Italian language fluency. The final sample consisted of 194 patients with end-stage cancer. Clinical and sociodemographic characteristics of the patients are reported in Table 3.

**Table 3.** Clinical and sociodemographic characteristics of patients’ sample (*N* = 194)

Characteristics	*n* (%)	Mean ± SD
Sex		
Male	88 (45.4)	
Female	106 (54.6)	
Age		64.18 (14.53)
Site		
Hospital	112 (57.7)	
Hospice	82 (42.3)	
Education		
Primary school	13 (6.7)	
Middle school	54 (27.8)	
High school	101 (52.1)	
Graduate	26 (13.4)	
Marital status		
Married	70 (36.3)	
Single	34 (17.6)	
Divorced	33 (17.1)	
Widow (er)	30 (15.5)	
Living with partner	26 (13.5)	
Employment status		
Employed	59 (30.4)	
Unemployed	8 (4.1)	
Retired	114 (58.8)	
Homemaker	10 (5.2)	
Cancer site		
Respiratory	33 (17.0)	
Gastrointestinal	39 (20.1)	
Hepatic–pancreatic	23 (11.9)	
Breast-gynecological	30 (15.5)	
Other	69 (35.6)	
Cancer stage		
Local	13 (6.7)	
Loco-regional	104 (53.9)	
Metastatic	76 (39.4)	
Religious practice		
Catholic	155 (79.9)	
Atheist	31 (16.0)	
Other	8 (4.1)	
Disease diagnosis and prognosis awareness		
No diagnosis, no prognosis	26 (13.4)	
Diagnosis, not aware of prognosis	37 (19.1)	
Diagnosis, prognosis overestimation	44 (22.7)	
Prognosis, no diagnosis	23 (11.9)	
Full awareness	64 (33.0)	
KPS		35.31 (11.39)
AKPS		34.02 (10.15)

SD = standard deviation; KPS = Karnofsky Performance Status; AKPS = Australian Modified Karnofsky Performance Status.

Frequencies of PeSAS scores are grouped into severe (≥8) and moderate (≥4 ≤ 7), alongside clinically significant (≥4). Frequency total scores are displayed in Table 4.

**Table 4.** Frequency of psycho-existential symptoms among palliative care patients (*N* = 194) in clinically significant (≥4), moderate 4–7 and severe (≥8) categories

Individual PeSAS symptoms	Severe ≥8% (95% CI)	Moderate 4–7% (95% CI)	Clinically significant ≥4% (95% CI)
Anxiety	14.4 (9.5–19.4)	42.8 (35.8–49.8)	57.2 (50.3–64.2)
Discouragement	20.6 (14.9–26.3)	41.2 (34.3–48.2)	61.9 (55–68.7)
Trapped by illness	28.4 (22–34.7)	50.5 (43.5–57.6)	78.9 (73.1–84.6)
Hopelessness	12.4 (7.7–17)	33 (26.4–39.6)	45.4 (38.4–52.4)
Pointlessness	11.9 (7.3–16.4)	35.6 (28.8–42.3)	47.4 (40.4–54.5)
Loss of control	8.8 (4.8–12.7)	41.2 (34.3–48.2)	50 (43–57)
Loss of roles	8.8 (4.8–12.7)	28.9 (22.5–35.2)	37.6 (30.8–44.5)
Depression	12.4 (7.7–17)	36.1 (29.3–42.8)	48.5 (41.4–55.5)
Wish to die	4.1 (1.3–6.9)	5.2 (2–8.3)	9.3 (5.2–13.4)
Confusion	6.2 (2.8–9.6)	14.4 (9.5–19.4)	20.6 (14.9–26.3)

CI = confidence interval.

**Figure 1.**
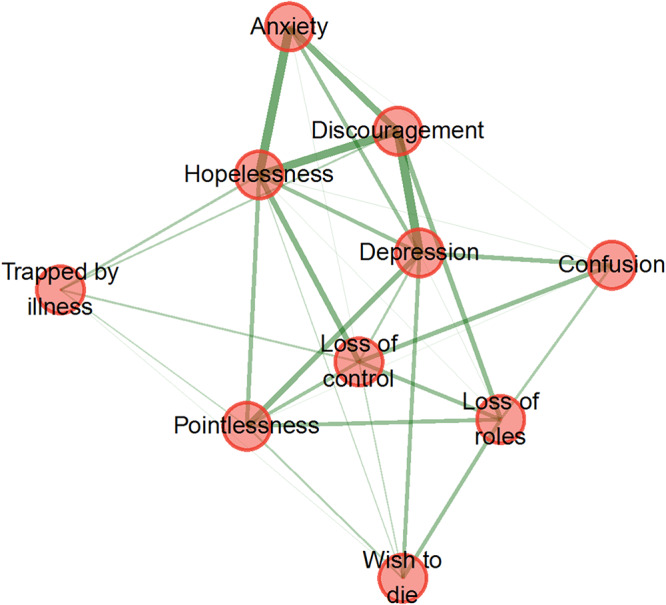
Exploratory graph analysis of psycho-existential symptoms assessed in 194 palliative care patients upon admission to a clinical service.

To confirm the discriminatory ability of PeSAS, patients with high PESAS scores (≥75th percentile = 48) had lower AKPS scores (*M* = 32.2, SD = 9.54) than patients with lower PeSAS scores (*M* = 35.0; SD = 10.17), but there was no statistical significance (*t*-test *p* = 0.082, *d* = −0.28). EGA was used to examine the structure of psycho-existential symptoms (Figure 1).

Depression (1.18) and discouragement (0.84), while being trapped by illness (−1.64), loss of roles (−0.17), and wishing to die (−1.12) showed the lowest strength values. A median network derived from 10,000 bootstrap iterations closely resembled the original network, supporting the stability of the observed structure. Centrality indices confirmed the role of hopelessness and depression as key nodes in the network. Hopelessness showed a value of 1.39 for both closeness and betweenness metrics, while depression had a closeness score of 1.23 and a betweenness score of 2.31.

**Figure 2.**
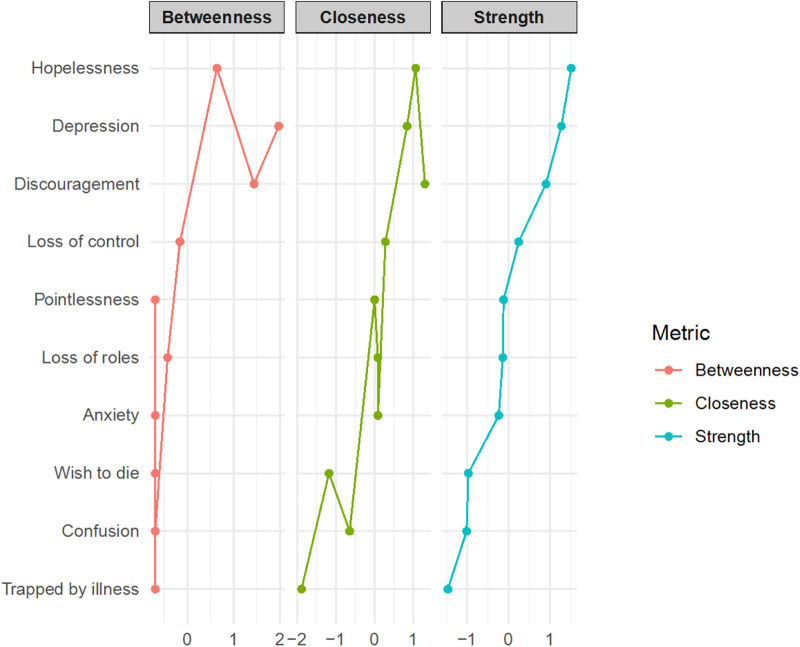
Standardized node centrality indices (*z*-scores) for PeSAS items.

Figure 2 presents centrality indices of the PeSAS items in the network.

## Discussion

This study aimed to assess the implementation of the PeSAS in palliative care, where HCPs had limited familiarity with psycho-existential distress screening. Experiential training workshops were conducted to improve clinicians’ competence in identifying and addressing psycho-existential symptoms in end-of-life cancer patients. Furthermore, we analyzed the frequency of psycho-existential symptoms assessed by the PeSAS and explored its structure using a network analysis.

The findings provided valuable insights into the role of PeSAS in clinical practice and highlight differences from previous research. Through role-play exercises, participants reported an increase in their confidence in symptom assessments, particularly suicidal ideation. They valued the structured methodology, which entails initial screening, targeted in-depth assessment, and a symptom management plan. This stepwise approach was perceived as practical and effective in facilitating the timely identification and management of psycho-existential distress. The overall effect of the training was moderate, suggesting an improvement in clinicians’ self-perceived. However, improvement was smaller than that reported by Kissane et al. (2022). This discrepancy may reflect differences in training intensity and duration, as well as sample characteristics. Contextual elements related to organizational culture, clinicians’ baseline familiarity with existential issues, beliefs, or emotional burden and burnout, could have influenced engagement with the training and its perceived effectiveness (Kissane et al. 2023). In the Italian healthcare context, lower outcomes on the PeSAS may reflect HCPs’ limited familiarity with and infrequent use of existential assessment tools, and a broader tendency among oncology and palliative care clinicians to refrain from systematically exploring such needs. Qualitative research confirms that clinicians often avoid existentially focused conversations due to fears of increasing patients’ burden and perceived gaps in their own skills for managing such sensitive discussions (Michael et al. 2025). Reluctance may also stem from moral distress, which can exacerbate avoidance of end-of-life conversations, as professionals fear both harming the patient and confronting their own vulnerability (Corradi-Perini et al. 2021).

Training interventions have shown effectiveness in enhancing clinicians’ confidence, but extended training and follow-up supervision are 2 potentially beneficial strategies. Research shows that while initial training can improve confidence, ongoing support is crucial for consolidating outcomes (Edmunds et al. 2013). This aligns with findings that supervision serves educational, supportive, and managerial functions in clinical practice (Rees et al. 2020), helping clinicians gain familiarity with assessing psycho-existential distress and improving their use of specific tools such as the PeSAS.

Implementation strategies were adapted to each site and tailored to the local challenges. Implementation strategies were adapted to each site and tailored to local challenges. When comparing services, it was observed that the implementation of the psycho-existential component progressed more rapidly in settings that included psychosocial staff, who tend to approach care with a focus on the person rather than purely on the disease (Bovero et al. 2020). In contrast, progress was slower in services operating within a strictly biomedical model of care, where psycho-existential aspects received less emphasis.

In line with Kissane et al. (2022), our results highlighted that psychosocial staff and a supportive service culture are key to sustaining psycho-existential screening, while structural barriers and biomedical focus can hinder it. Beyond technical training, a shift toward holistic, person-centered care is essential.

Comparison with Kissane’s study (2022) revealed similarities and differences in the frequency of psycho-existential symptoms, consistent with previous studies (Gan et al. 2022; Morita et al. 2004; Robinson et al. 2015; Tang et al. 2015; Tecuta et al. 2015). In both studies, feeling trapped by illness was the most frequently reported (78.9% in our sample compared to 53.7% in Kissane et al. (2022) suggesting that the perception of being physically and emotionally confined by illness plays a central role in existential suffering (Bovero et al. 2019). The higher frequency in our study may reflect a greater emphasis on autonomy and inability to cope as a distressing factor (Bovero et al. 2019). In advanced illness, loss of independence and progressive decline can foster a profound sense of being imprisoned by the disease (Bovero et al. 2024).

Discouragement also emerged as a key symptom. Literature has shown that early disheartenment plays a pivotal role in demoralization syndrome, closely associated with end-of-life existential distress (Gan et al. 2022).

Anxiety affected more than 57% of participants, while loss of control and depression were reported by approximately half of the patients. Interestingly, Hopelessness and Pointlessness appeared more prevalent in our sample. These findings may underscore the frequency and clinical significance of such symptoms at the end of life and support the need for routine, structured assessment using tools like the PeSAS.

In Kissane et al. (2022), the Wish to die was rated as severe in 7.6% of cases and moderate in 9.4%, reflecting findings consistent with broader clinical practice. In our study, the wish to die was rated as severe in 4.1% of the sample. This lower frequency may be influenced by cultural and healthcare contextual factors. The strong involvement of family caregivers, the cultural impact of Catholic traditions emphasizing the sanctity of life, and a palliative care model oriented toward relational and existential support may all contribute to mitigating the intensity of such wishes. Furthermore, assessments are often conducted after symptom management strategies have been implemented, potentially reducing the severity of wish-to-die expressions compared to other settings. Nevertheless, the presence of a severe wish to die, even in a minority, highlights the critical need for appropriate psychosocial interventions (Lu et al. 2024). This underscores the need for targeted psychosocial and spiritual interventions that help patients regain a sense of agency and meaning, even in the face of irreversible decline (Saracino et al. 2019).

Kissane et al. (2022) found a significant association between PeSAS scores and functional status (AKPS), whereas our findings did not, suggesting that patients may experience existential distress at any illness stage, as existential stressors can be present throughout the disease trajectory (Vehling et al. 2012).

Network analysis can reveal population-specific patterns in psycho-existential distress through symptom comparison, unlike Kissane et al. (2022), who identified Hopelessness, Pointlessness, and Trapped by illness as central – emphasizing Pointlessness as a driver of the wish to die – whereas our network showed a core cluster comprising Hopelessness, Discouragement, and Depression. Pointlessness and Trapped by illness were more peripheral, connected to the core primarily via Loss of control, a bridge to the central demoralization cluster. Moreover, Trapped by illness, Loss of roles, and wish to die exerted weaker influence than in Kissane’s model, suggesting that the prominence and connectivity of existential symptoms vary with patient population and disease context.

The prominence of Discouragement, Hopelessness, and Depression in our network likely reflects their shared role as core elements of demoralization and depressive states in advanced illness, including loss of meaning, diminished coping capacity, and a bleak outlook (Kissane et al. 2004; Vehling and Kissane 2018). In terminal cancer patients, progressive physical decline, loss of functional independence, and cumulative symptom burden can exacerbate psychological distress, reinforcing the interconnections among these symptoms and positioning them at the network’s core. Consistent with prior studies, hopelessness often serves as a “bridge” that connects affective and existential domains. Discouragement may act as an affective precursor, representing an early emotional response that precedes – and potentially contributes to – the development of hopelessness by signaling emotional struggles. Depression, in turn, may represent a broader syndrome integrating both affective distress and existential despair (Vehling et al. 2012; Robinson et al. 2015; Belvederi Murri et al. 2020). This result may contrast with the network presented by Kissane et al. (2022), where Depression was less central. These findings suggest that in terminal cancer, the interplay between physical decline, existential loss, and affective distress may strengthen the coupling of depressive and demoralization-related symptoms. EGA enhances our understanding of which symptoms occupy central positions. By identifying these central nodes, clinicians can prioritize intervention targets with the greatest potential to disrupt and ameliorate the cluster of related symptoms. This approach is particularly valuable in the context of psycho-existential distress, where targeting core symptoms – such as hopelessness or loss of meaning – through interventions like meaning-centered therapy or DT may produce broader improvements across the symptom network, thereby enhancing patient well-being and quality of life.

This study had several limitations. First, the sample was smaller than that used by Kissane et al. (2022), which may have reduced statistical power to detect effects. Second, the training format may require refinement to maximize its impact on clinicians’ confidence and competence. Third, although the network analysis provided valuable insights into the structure of psycho-existential symptoms, longitudinal data are needed to clarify how relationships between symptoms evolve over time.

Future research should refine and extend training, include follow-up assessments and real-case discussions, and explore cultural and setting-specific factors influencing the expression of psychosocial distress. Longitudinal studies could help map symptom trajectories and evaluate whether certain symptoms predict poorer outcomes.

## Conclusion

This study supports the clinical utility of the PeSAS in assessing psycho-existential distress and highlights the importance of targeted interventions addressing central symptoms such as hopelessness, discouragement, and depression. A strength of this work is its comprehensive description of an implementation program in palliative care, contributing to a field where systematic approaches to improving the quality of care for psycho-existential distress have rarely been explored. The findings demonstrate that clinicians can be trained to conduct structured screening for psycho-existential distress. Integrating such screening into routine practice can foster earlier detection, promote communication, and ensure psycho-existential suffering receives appropriate attention within comprehensive palliative care.

## Supporting information

10.1017/S1478951525101302.sm001 Bovero et al. supplementary material Bovero et al. supplementary material

## Data Availability

Data are available on request from the corresponding author.

## Supplementary material

The supplementary material for this article can be found at https://doi.org/10.1017/S1478951525101302.
