# Supplementary material for: Implementation of psycho-existential symptom distress screening among Italian healthcare providers
Source: Palliat Support Care. 2025 Dec 29;24:e8. doi: 10.1017/S1478951525101302 (PMC13166672; doi:10.1017/S1478951525101302)
Supplement: Bovero et al. supplementary material [file S1478951525101302sup001.docx]

| **Valutazione del benessere psico-esistenziale** | | | | Cognome:  Nome:    Data di nascita:  Diagnosi:  AKPS: | | | | | |
| --- | --- | --- | --- | --- | --- | --- | --- | --- | --- |
| **Scala valutazione sintomi**  La preghiamo di utilizzare questo modulo per informarci dei sintomi che la preoccupano, turbano o causano disagio. Queste informazioni ci aiuteranno a soddisfare le sue esigenze.  1. Scriva giorno o data nella prima riga        **Assente**    **Lieve**    **oderato**  **M**    **Grave**               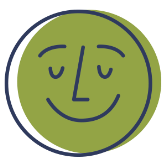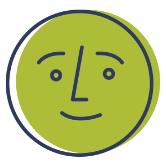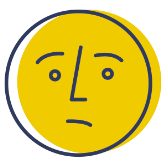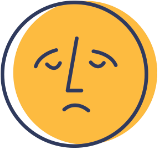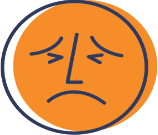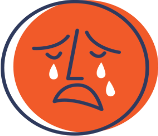                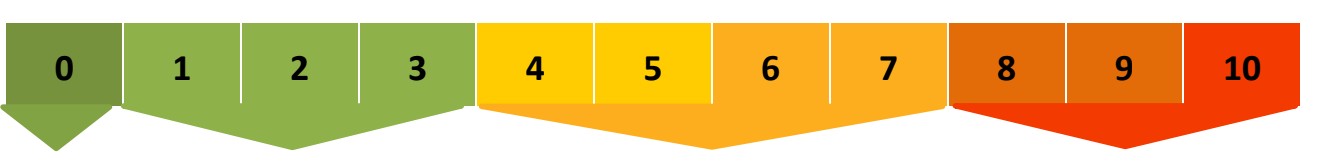 2. Usi la scala qui sopra per scegliere un numero da 0 a 10 che mostri quanto è preoccupato, turbato o in difficoltà | | | | | | | | | |
| **Giorno o data** |  |  |  |  |  |  |  |  |  |
| Ansia |  |  |  |  |  |  |  |  |  |
| Sconforto |  |  |  |  |  |  |  |  |  |
| Limitazione dalla malattia |  |  |  |  |  |  |  |  |  |
| Disperazione |  |  |  |  |  |  |  |  |  |
| Inutilità |  |  |  |  |  |  |  |  |  |
| Perdita di controllo |  |  |  |  |  |  |  |  |  |
| Perdita di ruoli |  |  |  |  |  |  |  |  |  |
| Depressione |  |  |  |  |  |  |  |  |  |
| Desiderio di morte |  |  |  |  |  |  |  |  |  |
| Confusione |  |  |  |  |  |  |  |  |  |

**PeSAS Sinonimi**

Sinonimi simili ai sintomi psico-esistenziali sotto osservazione.

| **PeSAS sintomi** | **Sinonimi semplici^*^** |
| --- | --- |
|  |  |
| **Ansia** | Preoccupazione, nervosismo, agitazione, inquietudine |
| **Sconforto** | Morale basso, demoralizzazione, mancanza di fiducia in sé stessi |
| **Limitazione** **dalla malattia** | Sentirsi bloccati, sentirsi intrappolati |
| **Disperazione** | Non vedere un futuro, sentirsi pessimisti |
| **Inutilità** | Non significato della vita, non avere uno scopo, non dare valore alla vita |
| **Perdita** **di controllo** | Sentirsi impotenti, non riuscire a pianificare |
| **Perdita** **di ruoli** | Partner, genitore, lavoro, perdita d’identità o di autostima |
| **Depressione** | Tristezza, umore depresso, mancanza di interesse, piacere o gioia |
| **Desiderio di** **morte** | Non voler continuare, voler farla finita, senza volontà di vivere, tendente al suicidio |
| **Confusione** | Sentirsi disorientati, sentirsi deliranti, sentirsi confusi riguardo alle cose |

^*^I sinonimi semplici sono 2-3 parole che possono essere ripetute dopo aver menzionato un sintomo per aumentarne la comprensione in caso ci sia difficoltà a comprendere ciò che viene chiesto da parte del paziente. Anche se esistono molti sinonimi che potrebbero essere utilizzati, questi sembrano risultare molto utili per la maggior parte dei pazienti.
